# Immunomodulatory Effects of SGLT2 Inhibitors—Targeting Inflammation and Oxidative Stress in Aging

**DOI:** 10.3390/ijerph20176671

**Published:** 2023-08-29

**Authors:** Ema Schönberger, Vjera Mihaljević, Kristina Steiner, Sandra Šarić, Tomislav Kurevija, Ljiljana Trtica Majnarić, Ines Bilić Ćurčić, Silvija Canecki-Varžić

**Affiliations:** 1 Department of Endocrinology, University Hospital Osijek, 31000 Osijek, Croatia; ema.schonberger@kbco.hr (E.S.); steiner.kristina@kbco.hr (K.S.); canecki.silvija@kbco.hr (S.C.-V.); 2 Faculty of Medicine, Josip Juraj Strossmayer University of Osijek, 31000 Osijek, Croatia; 3 Department of Pharmacology and Biochemistry, Faculty of Dental Medicine and Health, Josip Juraj Strossmayer University of Osijek, Josipa Huttlera 4, 31000 Osijek, Croatia; vnincevic@mefos.hr; 4 Department for Cardiovascular Disease, University Hospital Osijek, 31000 Osijek, Croatia; makarovic.sandra@kbco.hr; 5 Department of Internal Medicine and History of Medicine, Faculty of Medicine, Josip Juraj Strossmayer University of Osijek, Josipa Huttlera 4, 31000 Osijek, Croatia; 6 Department of Family Medicine, Faculty of Medicine, Josip Juraj Strossmayer University of Osijek, Josipa Huttlera 4, 31000 Osijek, Croatia; tkurevija@mefos.hr (T.K.); ljiljana.majnaric@mefos.hr (L.T.M.); 7 Health Center Osjecko-Baranjska County, 31000 Osijek, Croatia; 8 Department of Pharmacology, Faculty of Medicine, Josip Juraj Strossmayer University of Osijek, Josipa Huttlera 4, 31000 Osijek, Croatia; 9 Department of Pathophysiology, Faculty of Medicine, Josip Juraj Strossmayer University of Osijek, Josipa Huttlera 4, 31000 Osijek, Croatia

**Keywords:** inflammation, oxidative stress, SGLT2 inhibitors, aging, signaling pathways

## Abstract

Given that the increase in the aging population has grown into one of the largest public health issues, inflammation and oxidative stress, which are closely associated with the aging process, became a focus of recent research. Sodium-glucose co-transporter 2 (SGLT2) inhibitors, a group of drugs initially developed as oral antidiabetics, have shown many beneficial effects over time, including improvement in renal function and cardioprotective effects. It has been shown that SGLT2 inhibitors, as a drug class, have an immunomodulatory and antioxidative effect, affecting endothelial function as well as metabolic parameters. Therefore, it is not surprising that various studies have investigated the potential mechanisms of action of SGLT2 inhibitors in age-related diseases. The proposed mechanisms by which SGLT2 inhibitors can achieve their anti-inflammatory effects include influence on AMPK/SIRT1/PGC-1α signaling, various cytokines, and the NLRP3 inflammasome. The antioxidative effect is related to their action on mitochondria and their influence on the signaling pathways of transforming growth factor β and nuclear erythroid 2-related factor 2/antioxidant response element. Also, SGLT2 inhibitors achieve their anti-inflammatory and antioxidative effects by affecting metabolic parameters, such as uric acid reduction, stimulation of ketogenesis, reduction of body weight, lipolysis, and epicardial fat tissue. Finally, SGLT2 inhibitors display anti-atherosclerotic effects that modulate inflammatory reactions, potentially resulting in improvement in endothelial function. This narrative review offers a complete and comprehensive overview of the possible pathophysiologic mechanisms of the SGLT2 inhibitors involved in the aging process and development of age-related disease. However, in order to use SGLT2 inhibitor drugs as an anti-aging therapy, further basic and clinical research is needed to elucidate the potential effects and complex mechanisms they have on inflammation processes.

## 1. Introduction

One of the main causes of aging is low-grade inflammation. The term “inflammaging” describes the progressive and systemic development of a sterile, pro-inflammatory state that occurs during the aging process and can be attributed to a variety of possible causes [1,2]. Inflammaging is now considered an important factor in aging and is a major contributor to the occurrence of almost all age-related diseases. Therefore, growing interest in the field of anti-aging therapy is no surprise.

**Figure 1:**
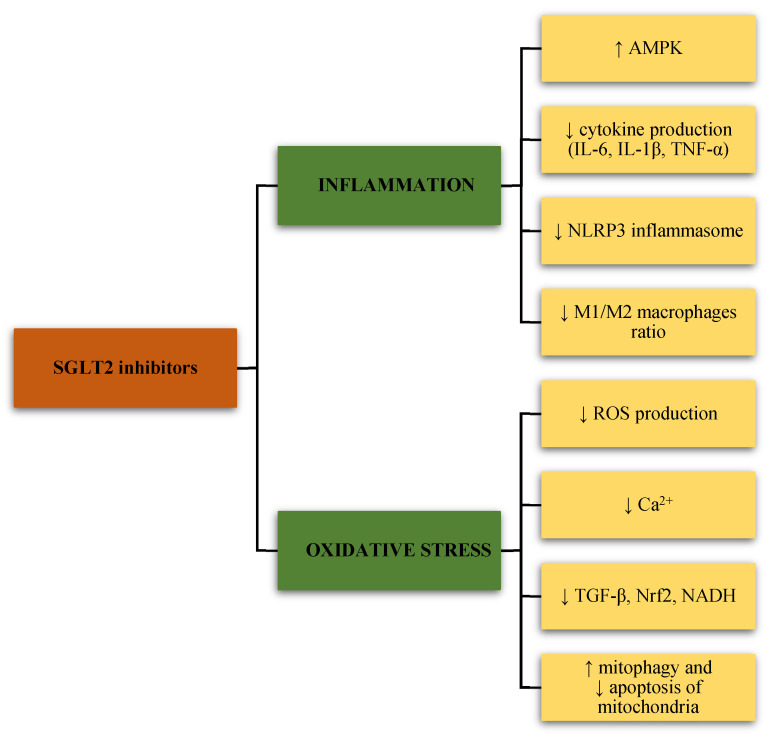
The effects of sodium-glucose cotransporter-2 (SGLT2) inhibitors on inflammation and oxidative stress. SGLT2, sodium-glucose cotransporter-2; AMPK, adenosine monophosphate-activated protein kinase; IL-6, interleukin-6; IL-1β, interleukin-1 beta; TNF-α, tumor necrosis factor-alpha; NLRP3, nod-like receptor (NLR) family pyrin domain containing 3; ROS, reactive oxygen species; Ca^2+^, calcium; TGF-β, transforming growth factor β; Nrf2, nuclear erythroid 2-related factor 2; NADH, nicotinamide adenine dinucleotide (NAD) hydrogen.

Sodium-glucose co-transporter 2 (SGLT2) inhibitors have emerged as a novel category of blood glucose-lowering drugs in clinical recommendations for a wide range of diseases [3,4,5,6]. The SGLT2 inhibitors empagliflozin and dapagliflozin better vascular function and avert vascular aging by decreasing the reactive oxygen species (ROS) content and increasing nitric oxide (NO) bioavailability, respectively [7]. It was discovered that ipragliflozin has the ability to prevent dysfunction of the endothelium, and this effect was connected with oxidative stress [8]. According to published data, SGLT2 inhibitors may delay vascular aging and arrest the development of endothelial dysfunction in animal models of type 2 diabetes (T2D) by reducing inflammation, oxidative stress, and glucose toxicity and increasing the survival of hyperglycemic endothelial cells [9]. The proposed anti-inflammatory and antioxidative effects of SGLT2 inhibitors are illustrated in Figure 1. Additionally, through its direct action on miRNA, the SGLT2 inhibitor dapagliflozin may enhance vascular functions and slow down the aging of blood vessels in diabetics. This is achieved through an increase in the expression of miR30e-5p and a decrease in the expression of miR199a-3p [10]. Overall, the data underline that SGLT2 inhibitors may improve vascular stiffness and aging, reduce inflammation and oxidative stress, delay endothelial and smooth muscle cell aging, regulate microRNA production, and potentially stop the occurrence and progression of atherosclerosis [7].

Interleukin (IL)-6 is presumably the most extensively researched cytokine among the numerous pro-inflammatory mediators that increase with aging and are related to outcomes associated with aging [11]. In fact, numerous cohort studies have shown an association between circulating IL-6 levels and the incidence of diabetes, cardiovascular events, all-cause mortality, and other disease related to aging [12,13]. The term “cytokine for gerontologists” (IL-6) was coined as a result of the available evidence. In three different studies, SGLT2 inhibition was shown to lower IL-6 levels [14,15,16].

Furthermore, SGLT2 inhibition has been shown to improve renal outcomes, all-cause mortality, and heart failure-related outcomes in people without diabetes [17,18]. Such observations could be supported by the tantalizing theory that the beneficial effect of SGLT2 inhibition is mainly caused by mimicking caloric restriction [19]. Given that aging is the main contributory factor for all of these illnesses [20], SGLT2 inhibitors could promote healthy longevity and could be investigated as a possible method to postpone age-related diseases.

The mammalian target of rapamycin (mTOR), adenosine 5′-monophosphate-activated protein kinase (AMPK), and sirtuin-1 (SIRT1) systems are important and interconnected modulators of aging and are among the most important nutrient sensors [21]. The regulation of mTOR activity, which is influenced by diet and hormones and is linked to aging, involves the formation of two complexes known as mTOR complex 1 (mTORC1) and mTOR complex 2 (mTORC2). Via phosphoinositide 3-kinase (PI3K) and protein kinase B (AKT) signaling, insulin and growth factors promote mTORC1, while AMPK has an inhibitory effect. AMPK, a sensor of the cell’s energetic state, is activated in response to an increase in the adenosine monophosphate (AMP)/adenosine triphosphate (ATP) ratio and is a key player in controlling the body’s overall energy balance [22]. SIRT1, a nicotinamide adenine dinucleotide (NAD)-dependent deacetylase that controls a variety of metabolic processes, can also be activated by AMPK [23]. There is a growing body of evidence supporting the beneficial effects of SGLT2 inhibitors on the mTOR, AMPK, and SIRT1 pathways in various tissues and cells, including kidney, pancreas, hepatocytes, cardiac microvascular endothelial cells, cardiomyocytes, and adipocytes [24,25,26,27,28,29,30].

A systematic review and meta-analysis of cardiovascular outcome studies, which assessed how SGLT2 inhibitors affected the incidence of major cardiovascular events (MACEs) in T2D patients categorized by age, found that age has no effect on the effectiveness profile of SGLT2 inhibitors versus placebo, with a hazard ratio (HR) of 0.83 (95% confidence interval [CI], 0.71–0.96) for those ≥65 years [31]. Combined analyses of phase III studies indicated that empagliflozin [32], dapagliflozin in individuals aged 65 years or older and 75 years or older [33], canagliflozin in patients aged 75 years or older [34], and ertugliflozin in individuals aged 65 years and above [35], had generally favorable safety profiles. Post hoc analyses of various cardiovascular outcome trials, including the EMPA-REG OUTCOME with empagliflozin [36], DECLARE-TIMI 58 with dapagliflozin [37], and VERTIS CV with ertugliflozin [38], revealed that SGLT2 inhibitors generally had the same efficacy and safety profile across different age ranges (<65 years vs. ≥65 to <75 years, and ≥75 years). The goal of EMPA-ELDERLY, the first randomized clinical study of an SGLT2 inhibitor in elderly T2D patients, was to increase the evidence supporting the use of SGLT2 inhibitors while also assessing the impact on muscle mass and muscle strength [39]. Some controversies are associated with the usage of SGLT2 inhibitors, which should be considered, especially in the elderly population. For instance, in The Canagliflozin Cardiovascular Assessment Study (CANVAS) and the Canagliflozin Cardiovascular Assessment Study-Renal (CANVAS-R), administration of canagliflozin was linked to a higher number of lower limb amputations compared to the placebo [40]. A recently published meta-analysis investigating a connection between weight and blood pressure reduction with lower limb outcomes demonstrated that a greater lowering of weight and blood pressure was associated with a higher risk of lower limb amputations and peripheral artery disease. The proposed mechanism includes enhanced diuresis caused by SGLT2 inhibitors, provoking volume loss and, subsequently, a decrease in tissue perfusion, leading to tissue necrosis [41]. Further clinical randomized trials and mechanistic studies are needed to clarify this potential risk connected with SGLT2 inhibitors, particularly in high-risk populations.

The other known adverse events are acute renal injury [42], genitourinary infection [43], hypotension [44] and ketoacidosis (although rare in T2D) [45]. In addition, a slightly higher incidence of bone fractures was observed with canagliflozin compared to the placebo (number of fractures in 1.5% vs. 1.1% of patients) [46]. There are still missing randomized controlled trials assessing the effectiveness and safety of SGLT2 inhibitors in the very old population, particularly in patients older than 75 years [47].

## 2. Effects of SGLT2 Inhibitors on Inflammatory Signaling Pathways

The AMPK molecule plays a vital role in the regulation of bioenergy metabolism and is pivotal in our understanding of diabetes mellitus and other metabolic disorders [48]. Over the past decade, data that AMPK is a critical regulator of the metabolic pathways that are involved in controlling inflammation has been rapidly accumulating [49]. AMPK serves as an energy sensor necessary for the management of inflammation in macrophages. It has been hypothesized that SGLT2 inhibitors may indirectly affect AMPK to reduce mTOR activity. Numerous studies have demonstrated that SGLT2 inhibitors can activate AMPK by restoring the AMP/ATP balance in favor of AMP, which is assumed to be the mechanism by which these medications have positive effects on the cardiac structure and microvessels, as seen in two separate mouse models [23]. Empagliflozin, a blood glucose-lowering drug, is involved in the energy metabolism process and could theoretically regulate AMPK and have anti-inflammatory effects that should be further investigated. At the gene level, empagliflozin reduces the expression of IL-6, IL-1β, tumor necrosis factor α (TNF-α) and monocyte chemoattractant protein-1 (MCP-1) mRNA in macrophages. It can induce an elevation in p-AMPK protein levels and a reduction in nuclear factor kappa B (NF-κB) protein levels within macrophages. These effects are observed as a result of ox-LDL exposure. By controlling AMPK signaling and thereby inhibiting NF-κB signaling, as seen in atherosclerotic plaque tissue and serum of studied mice, this drug has an anti-inflammatory effect. These discoveries offer new perspectives for therapeutic options in atherosclerotic diseases [50]. By reducing inflammation, oxidative stress, and atherosclerosis, both AMPK and SIRT1 have been shown to play essential roles in vasculature. AMPK activation may improve SIRT1 function by increasing the production of NAD +, which serves as an activator for SIRT1. It is generally established that, via modifying endothelial cell function, the AMPK/SIRT1 pathway represents a promising target for addressing vascular disorders associated with metabolism. According to current research, adiposity may decrease AMPK and SIRT1 activity in adipocytes, increase inflammatory responses and oxidative stress, and result in endothelial remodeling. In addition, in cell culture studies on human umbilical vein endothelial cells (HUVECs), high glucose (HG) caused the AMPK/SIRT1/PGC-1α axis to become inactive, as evidenced by decreased levels of SIRT1, peroxisome proliferator-activated receptor-gamma coactivator 1α (PGC-1α), and p-AMPK protein. However, dapagliflozin therapy restored SIRT1, PGC-1α, and p-AMPK in HG-treated HUVECs. These results suggest that AMPK/SIRT1 modulation is necessary for dapagliflozin to protect cells from endothelial injury induced by HG. Dapagliflozin partially regulates AMPK/SIRT1 signaling to protect against HG-induced endothelial injury. Moreover, dapagliflozin treatment reduced apoptosis, ROS, and inflammation in HG-induced endothelial cell failure via modulating the AMPK/SIRT1/PGC-1 signaling pathway [51].

Although the innate immune system can be chronically activated by a variety of its own and foreign molecules, there are few chemical pathways that have been proposed to cause inflammation [2]. The nod-like receptor (NLR) family pyrin domain containing inflammasome platform 3 (NLRP3) is an essential mechanism implicated in the age-related pro-inflammatory process. The NLRP3 inflammasome consists of the assembly of the multi-protein complex NLRP3-apoptosis-associated speck-like protein (ASC) with a C-terminal caspase recruitment domain (CARD). This complex facilitates the processing of pro-IL-1β and pro-IL-18 into their active forms by cleavage of caspase-1. The activation of the NLRP3 inflammasome can be triggered by various pathogens or cellular damage. Following treatment with SGLT2 inhibitors in heart and kidney tissue in mouse models, numerous studies have shown suppression of the NLPR3 inflammasome and decreased release of IL-1 [23]. The NLRP3 inflammasome is controlled by metabolism. Reactive oxygen species produced by mitochondria have been associated with the activation of NLRP3 [48]. Moreover, the endogenous inhibitor of NLRP3 activation is the ketone body β hydroxybutyrate (β-OHB), produced by fatty acid oxidation in the liver at low glucose. In addition, it has been proposed that fatty acid synthesis controls the activation of NLRP3 and the formation of IL-1β and IL-18. The clinical applications of the anti-inflammatory effects of SGLT2 inhibitors through immunomodulatory metabolites seem promising. Dapagliflozin blocked the activation of the NLRP3 inflammasome by promoting the metabolite itaconate from the mitochondrial TCA cycle in the chronic fibrosis kidney in mice models. Ye et al. demonstrated that dapagliflozin, an SGLT2 inhibitor, reduced NLRP3/ASC activation in samples of myocardial tissue in mice with T2D, slowing the progression of diabetic cardiomyopathy. Furthermore, a recent study found that dapagliflozin inhibited the NLRP3 inflammasome and activated AMPK, which protected against the progression of diabetic cardiomyopathy and cardiac fibrosis. Dapagliflozin also protected animals from steatosis, inflammation, and liver injury by preventing the activation of the ROS-NLRP3 inflammasome. A different study showed that dapagliflozin can treat atherosclerosis in diabetes by altering the ROS-NLRP3 caspase-1 pathway and preventing the release of IL-1 in aorta tissue [52]. From a mechanistic point of view, it has already been shown that switching from glycolysis to ketogenesis leads to the inactivation of immune cell inflammasomes and a reduction in immunopathology [53]. Consequently, this phenotype is likely to be reproduced by SGLT2 inhibitor-induced ketonemia [54]. Senescent cell accumulation and NLRP3 inflammasome activation, two processes considered major causes of aging, are inhibited by SGLT2 inhibition in many tissues. The findings suggest that several potential intermediary mechanisms, including the metabolic shift to ketonemia, reductions in insulin and uric acid levels, and potentially the direct involvement of SGLT2 in senescent cells, may contribute to these observations [23].

Depending on their degree of activation or polarization, tissue macrophages are classified as either M1 macrophages (classically activated, pro-inflammatory) or M2 macrophages (alternatively activated, anti-inflammatory). The M1/M2 polarization of macrophages is a very dynamic process, and under certain physiological and pathological circumstances, the phenotype of polarized macrophages can be reversed. M2-polarized macrophages produce anti-inflammatory cytokines, such as IL-10 and IL-1 receptor antagonists. However, in the adipose tissue of obese individuals, the production of these cytokines is diminished [55]. Empagliflozin reduces inflammation in high-fat obese (DIO) mice and attenuates the insulin resistance associated with obesity by polarizing M2 ATMs. By controlling macrophage recruitment and the M1/M2 status, empagliflozin plays a crucial role in addressing the adipose tissue inflammation and insulin resistance associated with obesity. Empagliflozin was able to decrease plasma TNF levels, suppress the accumulation of M1-polarized macrophages, induce the expression of the anti-inflammatory M2 phenotype in white adipose tissue (WAT) and liver macrophages, and prevent obesity-induced chronic inflammation in DIO mice [56]. Table 1 shows the potential anti-inflammatory effects of SGLT2 inhibitors in a preclinical setting.

## 3. Antioxidative Mechanisms and Mitochondria Protection of SGLT2 Inhibitors

Ever since the beginning of research regarding the physiology of aging, mitochondria have always been the focus of investigations. Due to its exceptional role in cell physiology, great efforts have been made to investigate its role in aging and, thus, the potential mechanisms enabling us to affect that process. There are many theories trying to explain the role of mitochondria in aging. One of the very first, suggested by D. Harman in the middle of the last century, is based on the accumulation of ROS and their negative pathophysiological effects [61]. Despite the emergence of new ones, Harman’s theory is still in focus, as confirmed by some recent research [62,63]. However, as a cause of mammalian mtDNA mutations, a recent study marked replication errors made by the mtDNA polymerase rather than originating from ROS [64]. Furthermore, as the accumulation of damaged and dysfunctional mitochondria in senescence are associated with increased disease emergence and aging, the disturbed process of timely displacement of such mitochondria, known as mitophagy, is becoming more popular [65,66]. Some other theories highlight the importance of mitochondrial sirtuins, interpreting them as cell stress sensors due to their NAD+ dependence linking their enzymatic activity to the cell metabolic state [67]. Finally, mitochondria play a crucial role in the inflammation process. This mechanism is based on the activation of immune cells caused by ROS and their recruitment into the tissue, which, combined with oxidative stress itself, creates a pro-inflammatory environment and leads to inflammation that ultimately causes the occurrence of chronic diseases and accelerates aging [68,69].

ROS, as a part of free radicals, including reactive nitrogen species (RNS) and reactive sulfur species (RSS), has a significant role in ion transportation, gene expression cell signaling, and apoptosis during functional activities of the cell and cell metabolism. These free radicals are small, highly reactive, and can cause deleterious effects on different molecules. Cells normally have a defense system against damage caused by ROS; however, in times of environmental stress and cell dysfunction, their amount significantly increases, and their effect on macromolecules causes oxidative stress [70,71]. As already mentioned, the human body has developed an antioxidant defense system in order to neutralize free radicals after their formation in the body. It includes free radical scavenging methods and enzymatic and metal ion chelating. To determine antioxidant activities, a model of direct scavenging of superoxide has been used. Superoxide is a dominant, cellular, free radical that is a by-product of mitochondrial respiration and different enzymes, including nicotinamide adenine dinucleotide (NAD) + hydrogen (NADH) oxidase, xanthine oxidase (XO), cyclooxygenases, and monooxygenases. Metal ion chelating usually refers to transition metals such as iron and copper. These metals react with hydrogen peroxide, which exacerbates oxidative stress. Also, in the prevention of deleterious effects of oxidative stress, modulation of NADPH oxidase has been proposed, as it is known that NADPH oxidases and XO are the main enzymes in the production of free radicals [70]. Antioxidants are substances that are highly useful due to reduction-oxidation reaction-related capabilities [72]. These substances may directly react with free radicals, neutralizing them, thus becoming less reactive and less dangerous radicals than the original ones [70]. Therefore, many studies are directed toward the development of medicines based on mitochondria-targeted antioxidants based on conjugates of plant alkaloids, amino-acid- and peptide-based compounds, and molecules conjugated with lipophilic cations or rhodamine and liposomes. However, it has been shown that such drugs have a questionable effect on neurodegenerative disease, cardiovascular disease, cancers, chronic obstructive pulmonary disease and Duchenne muscular dystrophy, and some of them even showed a toxic effect in humans [73].

On the other hand, by studying the in vitro and in vivo effects of the SGLT2 inhibitors, many positive effects of these drugs were noted, apart from their primary hypoglycemic effect, and some of them are attributed to their action on mitochondria. It has been shown that they can modulate the pathophysiologic pathways caused by the dysfunction of mitochondria, thus influencing the prevention of the development of complications of diabetes mellitus. The influence of SGLT2 inhibitors on mitochondrial function and oxidative stress can occur through various mechanisms (Figure 1). One of the effects of SGLT2 inhibitors is the reduction of mitochondrial ROS production and reduction of Ca^2+^ overload in mitochondria, affecting vascular function in the diabetic milieu. Such a reduction of oxidative stress affects the reduction of endothelial dysfunction and reduces the microvascular complications of diabetes mellitus in the myocardium. SGLT2 inhibitors can affect the reduction of the level of electron donors, such as NADH, by reducing the level of glucose.

Augmented NOX4 activity is also associated with elevated ROS production. In an ISO-induced oxidative stress model in mice, canagliflozin reduced NOX4 protein expression in the heart and kidneys [74,75]. In a study by Li et al., empagliflozin was demonstrated to significantly decrease the NOX4 levels in the myocardial tissue of diabetic rats, primarily by reducing NADPH oxidase activity and potentially ameliorating diabetic cardiomyopathy [76]. Furthermore, the study showed that empagliflozin could facilitate the translocation of nuclear erythroid 2-related factor-2 (Nrf2) to the nucleus, thereby activating Nrf2/antioxidant response element (ARE) signaling to modulate oxidative stress in the myocardium.

A recent study that addressed oxidative stress, platelet activation, and thrombus growth before and after 15 days of treatment with SGLT2 inhibitors showed a protective mechanism on thrombus formation in individuals with T2D. Patients further exhibited diminished oxidative stress through the lowering of NADPH oxidase 2 (NOX2) activity and H2O2 levels. Additionally, dapagliflozin demonstrated its capacity to restore platelet activation, as determined by reduced thromboxane formation and levels of soluble P-selectin and soluble CD40 ligand. These findings indicate that the potential cardiovascular protection offered by SGLT2 inhibitors might be attributed to their antiplatelet and antithrombotic activities [77].

**Figure 2:**
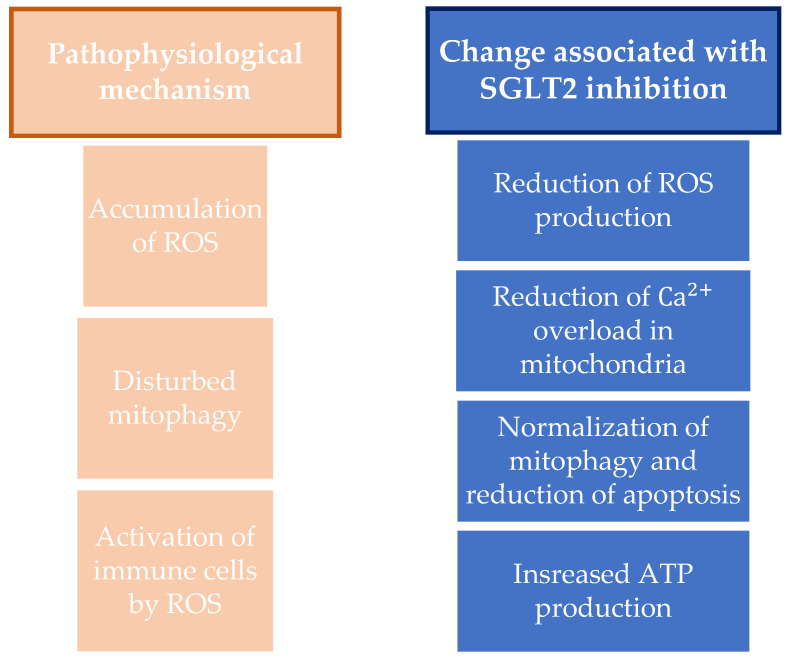
Summary on the antioxidative mechanisms and mitochondria protection and on changes associated with SGLT2 inhibition. ROS, reactive oxygen species; SGLT2, sodium-glucose cotransporter-2; ATP, adenosine triphosphate.

In addition, SGLT2 inhibitors affect the morphology and quantity of mitochondria, i.e., they normalize mitophagy and reduce the apoptosis mediated by the glucoregulation effect. By normalizing mitochondrial mitophagy and thereby preserving mitochondria, they have a cardioprotective effect and can reduce or delay the onset of complications of diabetes mellitus. This effect is achieved via the transcription factors PGC-1α and mitochondrial transcription factor A and by regulating mitochondrial fission via dynamin-related protein 1, mitofusin 1, and mitofusin 2. SGLT2 inhibitors also achieve their effect on mitochondria by increasing ATP production through the upregulation of genes that affect oxidative phosphorylation and fatty acid metabolism, influencing the modulation of mitochondrial ions and preserving the mitochondrial membrane potential. In addition, other potential mechanisms of action of the SGLT2 inhibitors on mitochondria are being studied [78,79]. A summary showing the mechanisms of oxidative stress and antioxidative effects of SGLT2 inhibitors is shown in Figure 2.

## 4. Effects of SGLT2 Inhibitors on Metabolic Parameters

### 4.1. Reduction of Uric acid

Uric acid, the end product of purine metabolism in the human body, has been associated with the development of cardiovascular (CV) disease [80,81,82], heart failure (HF) [83], metabolic syndrome [84], and chronic kidney disease [85,86]. Several different enzymes, such as XO, regulate serum uric acid. Elevated serum levels of uric acid could be a result of increased production and/or reduced elimination and have been associated with increased inflammation, oxidative stress, decreased NO production, and consequent endothelial dysfunction [87]. Both experimental and clinical trials support the argument that serum uric acid serves as a biomarker of oxidative stress rather than causing a heart injury. In patients with chronic HF, the level of XO in the myocardium is elevated, and therefore, uric acid is secreted from the failing heart [88].

SGLT2 inhibitors have been shown to reduce uric acid concentrations [89], and the previous assumption regarding the uric acid lowering effects of SGLT2 inhibitors was that they increase uric acid elimination in the kidney [90,91]. Filtered uric acid is reabsorbed in the proximal renal tubules, mostly via the urate transporter URAT1 and the glucose transporter 9 (GLUT9). By reducing the transport function of SGLT2 with SGLT2 inhibitors, the glucose concentration in the proximal renal tubule increases and competes with uric acid for GLUT9 in the basolateral membrane, resulting in increased uric acid excretion [84]. Moreover, glycosuria caused by SGLT2 inhibitors provokes a metabolic switch toward a fasting-like state, which activates SIRT1, a sensor that is activated by nutrient deprivation and whose primary role is to maintain blood glucose. SIRT1 is also a part of the cellular response to inflammatory and oxidative stressors, and its activation induced by SGLT2 inhibitors explains why they can ameliorate cardiac and renal injury [92]. SIRT1 activation in oxidative stress results in decreased XO activity and, consequently, diminished uric acid levels [93]. Additionally, SIRT1 renews the endothelial nitric oxide synthase (eNOS) capacity to produce NO. Therefore, the antioxidant effect of SGLT2 inhibitors might suggest why a lower uric acid concentration is a significant predictor of this drug-class effect on CV events reduction [88,94,95].

### 4.2. Stimulating Ketogenesis

**Figure 3:**
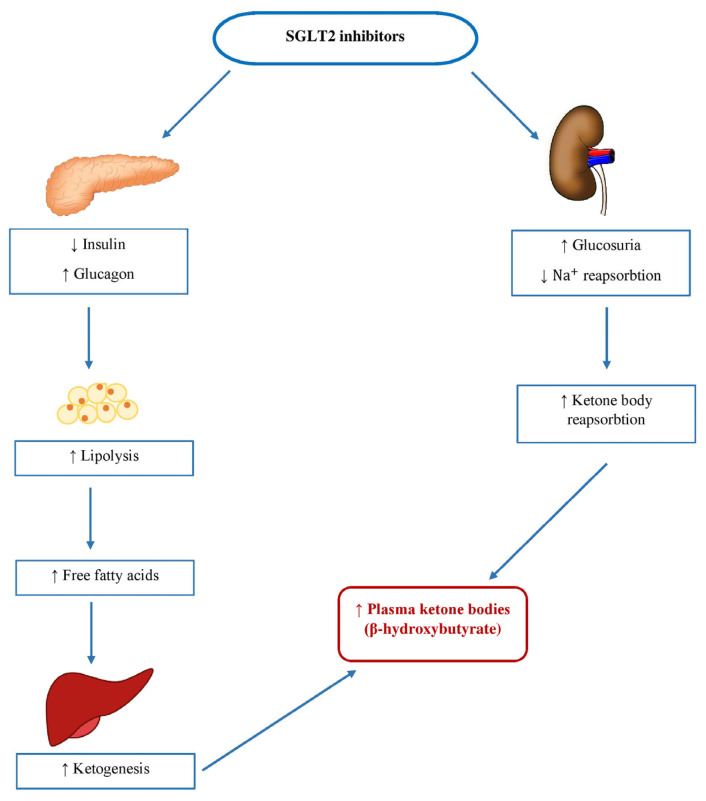
Mechanism of stimulating ketogenesis with sodium-glucose cotransporter-2 (SGLT2) inhibitors. SGLT2, sodium-glucose cotransporter-2; Na^+^, sodium.

In chronic diseases, such as HF and T2D, myocardiocytes’ usage of glucose is compromised; thus, most of the energy comes from free fatty acid (FFA) oxidation. This process causes increased oxygen uptake by the myocardiocytes, leading to increased ROS production, which can precipitate the development of diastolic dysfunction, further damaging already impaired heart function [96,97]. Given that ketone bodies generate more ATP per molecule of oxygen consumed compared to glucose or FFA, they represent a good source of energy, improving cardiac metabolic efficiency. Studies have suggested that the SGLT2 inhibitor empagliflozin induces glycosuria in patients with T2D, resulting in decreasing plasma glucose and insulin levels while increasing fasting and post-meal glucagon concentrations [98]. Due to a reduced insulin-to-glucagon ratio, FFA transport to the liver is enhanced, which promotes ketogenesis. Therapy with empagliflozin was linked to higher levels of circulating FFA and glycerol during fasting and after meals, as well as higher levels of plasma β-OHB, which were increased twofold to threefold [99] (Figure 3). β-OHB has been found in several human and animal studies to improve cardiac output and diastolic function [100]. In a Japanese phase III trial lasting 24 weeks and involving individuals with T2D who had not previously been treated with medication, the levels of ketones in the plasma increased proportionally to the dose when 100 mg or 200 mg of canagliflozin were administered, in comparison to a placebo, over the entire duration of the study. The changes from baseline to Week 24 were −12.5, +64.5, and +146.5 mg/dl in the placebo, 100 mg, and 200 mg groups, respectively [101]. Furthermore, another study showed an increase in fasting β-OHB levels after chronic treatment with empagliflozin, shifting it from an initial level of 246 ± 288 µmol/L to 561 ± 596 µmol/L (significant at *p* < 0.01) [102].

The NLRP3 inflammasome is a complex formed by multiple proteins in the cytoplasm of innate immune cells. In 2015, it was reported that β-OHB decreases the NLRP3 inflammasome’s activation and lowers the production of IL-1β in macrophages and mice [53]. A study by Kim et al. demonstrated that therapy with the SGLT2 inhibitor empagliflozin in individuals with T2D and a high CV risk attenuated NLRP3 inflammasome activation and the secretion of IL-1β [103]. At least partially, this was accomplished by raising the serum β-OHB levels, which may contribute to the antioxidant and anti-inflammatory effects of SGLT2 inhibitors.

### 4.3. Effects on Body Mass and Adipose Tissue

T2D, insulin resistance, metabolic syndrome, and an elevated risk of CV disease are all associated with obesity, particularly visceral adiposity [104]. Obesity is closely connected to chronic inflammation, characterized by aberrant cytokine production, elevated acute-phase reactants and other mediators, and activation of inflammatory signaling pathways [105]. Particularly, leptin appears to have a role in various obesity-related CV diseases, whereas adiponectin appears to have a cardioprotective role [106,107]. Changes in the structure and leptin metabolism affect the growth of epicardial adipose tissue (EAT) mass, which plays a major role in the development of HF due to cardiac fibrosis and inflammation [108] and is significantly correlated with the degree and severity of coronary artery disease [109]. EAT is composed of the adipose tissue depot located next to the myocardium, and excessive EAT contributes to the development of CV disease through the secretion of pro-inflammatory mediators by possible paracrine or endocrine effects [110]. Some studies have shown that SGLT2 inhibition can alter the secretion profile of adipokines, resulting in reduced serum leptin and increased adiponectin concentrations [111]. Dapagliflozin treatment was reported to reduce the EAT volume and plasma levels of TNF-α and plasminogen activator inhibitor-1 (PAI-1) [112]. In another study, it was demonstrated that canagliflozin decreased the serum concentrations of leptin and IL-6 compared to glimepiride but had no effect on TNF-α [16]. In patients undergoing heart surgery, dapagliflozin enhanced the EAT cell differentiation, increased glucose absorption, and decreased the release of pro-inflammatory cytokines, with a positive effect on the recovery of human coronary artery endothelial cells [113]. These observed changes may be a result of the systemic effects of SGLT2 inhibition, such as a loss of weight and increased lipolysis.

SGLT2 inhibitor administration is associated with the loss of approximately 200–250 kcal per day through increased urinary glucose excretion [114]. A meta-analysis of clinical studies on SGLT2 inhibitors indicated a typical weight loss of 2 kg in comparison to the placebo [115], and it is observed during the first weeks of treatment, reaches a plateau after six months [116], and may last for up to four years [117]. The observed reduction in body mass is far lower than what the negative calorie balance would predict. Ferrannini et al. hypothesized that this difference resulted from a compensatory rise in calorie intake that followed the start of treatment a few weeks later [118]. Studies using bioimpedance spectroscopy demonstrated that weight loss with the SGLT2 inhibitor treatment is mainly caused by a reduction in visceral and subcutaneous adipose tissue mass while maintaining lean tissue mass [104,119]. Table 2 shows the pathophysiological mechanisms mentioned in this chapter and how they are affected by SGLT2 inhibitor therapy.

## 5. Improving Endothelial Function

Studies have shown that endothelial dysfunction, which is primarily characterized by a decrease in the bioavailability of NO, is an early stage in the development of atherosclerosis. The overexpression of adhesion molecules and pro-inflammatory cytokines disrupt the normal ability of the endothelium to facilitate the dilation of blood vessels [120]. Clinical studies have shown that antidiabetic drugs have the ability to decrease the production and release of pro-inflammatory, atherosclerotic, and oxidative substances. Furthermore, they have been found to enhance flow-mediated dilation, indicating their favorable impact on endothelial function [8].

The vascular dysfunction observed in diabetic patients can be attributed to the presence of ROS generated in a hyperglycemic state. Increased blood sugar levels enhance ROS production through multiple pathways. The generated ROS hinders the phosphorylation of eNOSSer1177 by Akt, resulting in the disruption of the endothelium-dependent vasorelaxation mediated by NO. In an experiment involving induced oxidative stress through isoprenaline, eNOS phosphorylation was considerably hindered in the heart and kidneys. However, this inhibition was reversed by the administration of canagliflozin, and the restoration was impeded by an AMPK inhibitor, consistent with the influence of AMPK/Akt signaling on canagliflozin’s effect [74,75]. Similarly, other studies showed that treatment with ipragliflozin [8] and empagliflozin [121] increased the phosphorylation of eNOSSer1177 in the heart tissue of mice, indicating the protective effects on endothelial cells. Furthermore, ROS activates redox-sensitive transcription factors, such as NF-κB, triggering the expression of inflammatory molecules like intercellular adhesion molecule (ICAM)-1 and vascular cell adhesion molecule (VCAM)-1. These molecules promote interactions between the endothelium and leukocytes, expediting the progression of vascular inflammation [122,123,124]. Moreover, a study by Takahashi et al. demonstrated that the combined treatment of ipragliflozin and empagliflozin suppressed the proliferation of vascular smooth muscle cells (VSMCs) and the development of neointima after vascular damage in diabetic mice, suggesting the attenuation impact of neointima formation after vascular injury [125].

The administration of canagliflozin to obese mice for a duration of eight weeks resulted in a decrease in the expression of pro-inflammatory biomarkers, including ionized calcium-binding adaptor molecule 1 (Iba1), IL-6, and TNF-α, in the neural tissues. Additionally, it reduced macrophage infiltration in skeletal muscle. These findings indicate that inhibiting SGLT2 disrupts the harmful cycle of obesity and inflammation. The beneficial effects are not only attributed to promoting caloric loss but also involve the suppression of inflammation associated with obesity in both the nervous system and skeletal muscle [57]. Moreover, in a study by Han et al., administering empagliflozin for eight weeks demonstrated a reduction in the circulating pro-inflammatory markers like hs-CRP, TNF-α, IL-6, and MCP-1 in ApoE−/− mice fed a Western diet in addition to lowering their glucose levels and insulin resistance. There was a significant correlation between the decrease in inflammatory markers and the reduction in the size of atherosclerotic plaque in the aortic arch/valve [58]. The positive effects of dapagliflozin on atherosclerosis have been observed not only in mouse models but also in experiments involving other animal species. For instance, in a rabbit model of atherosclerosis, dapagliflozin demonstrated anti-atherosclerotic properties by regulating the inflammatory responses (resulting in a reduced expression of TNF-α, IL-1β, and IL-6) and promoting macrophage polarization towards M2 macrophages, even in non-diabetic conditions [59]. Ishibashi et al. conducted a study demonstrating that the use of tofogliflozin for 4 h and 24 h effectively decreased the production of ROS, expression of MCP-1, and occurrence of cellular apoptosis in proximal tubular cells derived from human kidneys that were subjected to high levels of glucose stimulation [60]. The mechanisms associated with endothelial dysfunction and the changes caused by SGLT2 inhibition are presented in Table 3.

## 6. Conclusions

The development of almost all age-related diseases, including CV diseases, T2D, and chronic kidney disease, is significantly influenced by the systemic pro-inflammatory state. Growing data highlights the significance of SGLT2 inhibitors in reducing inflammation and oxidative stress, positioning this drug class as a potential therapeutic option to postpone the occurrence of age-related diseases. The majority of published research connects these anti-inflammatory effects to SGLT2 inhibitors’ systemic and metabolic benefits. Even though it is based on a lesser number of studies so far, the claimed advantages in chronic diseases may partly be the result of the direct action on pro-inflammatory signaling pathways. SGLT2 inhibitors may indirectly affect the AMPK/SIRT1 pathway with consequent anti-inflammatory effects. Furthermore, SGLT2 inhibitors have shown suppression of the NLPR3 inflammasome and decreased release of IL-1, at least partially as a result of ketogenesis and increased serum β-OHB levels. It was demonstrated that SGLT2 inhibitors can potentially display anti-atherosclerotic properties via controlling inflammatory responses and macrophage polarization toward the anti-inflammatory M2 phenotype. As shown in this paper, SGLT2 inhibitors have numerous beneficial effects. In the long term, they reduce cardiovascular risk, thereby reducing the risk of heart failure, acute myocardial infarction and, ultimately, mortality. In addition to the above, they also have long-term effects on non-alcoholic fatty liver disease and are renoprotective. It is believed that the SGLT2 inhibitors’ effect on immunomodulation is responsible for part of these effects. Considering that the first SGLT2 inhibitor was approved by the FDA in 2013, time and further research are needed to demonstrate the long-term effects of SGLT2 inhibitors on age-related diseases [126,127,128,129]. The results of recent clinical trials involving SGLT2 inhibitors have shown therapeutic prospects and pharmacological mechanisms beyond SGLT2 inhibition and glycemic control. Therefore, the introduction of SGLT2 inhibitors should be considered as early as possible in the treatment of some of the most common chronic diseases in the elderly—chronic kidney diseases, T2D, and CV diseases. Still, careful assessment is necessary, given the possible adverse outcomes, especially in vulnerable populations. In conclusion, SGLT2 inhibitors may be promising therapeutic agents with pleiotropic effects on metabolic regulation and a reduction in CV and renal complications, while additional studies to understand its benefits on multiple organs affected by aging are required.

## Figures and Tables

**Table 1:** Selected preclinical studies on the anti-inflammatory effects of sodium-glucose cotransporter-2 (SGLT2) inhibitors depending on the agent and experimental model.

Study	SGLT2 Inhibitor	Experimental Setting	Findings
Salim et al. [8]	Ipragliflozin	Male C57BL/6 mice	↓ MCP-1, VCAM-1, ICAM-1, ROS and ↓ endothelium dysfunction in the abdominal aorta
Fu et al. [50]	Empagliflozin	Apoe−/− mice	↑ AMPK in aorta tree and aortic valve area
Faridvand et al. [51]	Dapagliflozin	HUVECs	↓ ROS, IL-6, TNF-α and ↑ SIRT1, PGC-1α, p-AMPK in HUVECs
Abd El-Fattah et al. [52]	Dapagliflozin	Sprague Dawley rats	↓ MCP-1, IL-1β, IL-18, TNF-α, ↓ NLRP3 levels and ↑ p-AMPK in lung tissue
Xu et al. [56]	Empagliflozin	C57BL/6J mice	↓ M1/M2 macrophage ratio in WAT and liver ↑ FGF-21 in liver and plasma ↑ AMPK in skeletal muscle
Kogot-Levin et al. [25]	Dapagliflozin	Diabetic Akita mice	↓ mTORC1 in renal proximal tubule cells
Meng et al. [27]	Empagliflozin	Male C57BL/6J mice	↑ AMPK/mTOR pathway in hepatic macrophages
Tian et al. [29]	Empagliflozin	H9c2 cells	↑ SIRT1/PTEN/Akt pathway in cardiomyocytes
Lee et al. [30]	Ipragliflozin	Male 129S6/Sv mice	↑ AMPK/SIRT1 pathway in liver and WAT
Cai et al. [28]	Empagliflozin	CMECs	↑ AMPKα1/ULK1 pathway in cardiac microvascular endothelial cells
Naznin et al. [57]	Canagliflozin	Male C57BL/6J mice	↓ Iba1, IL-6 and ↓ macrophage accumulation in skeletal muscle
Han et al. [58]	Empagliflozin	Male apoe−/− mice	↓ hs-CRP, TNF-α, IL-6 and MCP-1 in adipose tissue
Lee et al. [59]	Dapagliflozin	Male New Zealand white rabbits	↓ TNF-α, IL-6 and ↓ macrophage infiltration in the abdominal aorta
Ishibashi et al. [60]	Tofogliflozin	Cultured proximal tubular epithelial cells from human kidney	↓ MCP-1, ROS in human proximal tubular cells

SGLT2, sodium-glucose cotransporter-2; MCP-1, monocyte chemoattractant protein-1; VCAM-1, vascular cell adhesion molecule 1; ICAM-1, intercellular adhesion molecule 1; ROS, reactive oxygen species; AMPK, adenosine monophosphate-activated protein kinase; IL, interleukin; TNF-α, tumor necrosis factor α; HUVECs, human umbilical vein endothelial cells; SIRT1, sirtuin-1; PGC-1α, peroxisome proliferator-activated receptor-gamma coactivator 1α; NLRP3, nod-like receptor family pyrin domain containing 3; WAT, white adipose tissue; FGF-21, fibroblast growth factor 21; mTORC1, mammalian target of rapamycin (mTOR) complex 1; PTEN, phosphate and tensin homolog; Akt, protein kinase B; ULK1, Unc-51-like autophagy activating kinase 1; Iba1, ionized calcium-binding adaptor molecule 1; hs-CRP, high-sensitivity C-reactive protein.

**Table 2:** Summary of SGLT2 inhibitors’ effects on metabolic parameters.

Pathophysiological Mechanism	Change Associated with SGLT2 Inhibition
Increased production and/or reduced elimination of uric acid	Increased uric acid elimination in the kidney via GLUT9 Activation of SIRT1 → decreased XO activity
Increased FFA oxidation → increased ROS production	Enhanced FFA transport to the liver → stimulation of ketogenesis (higher plasma β-OHB)
Activation of NLRP3 inflammasome → processing of pro-IL-1β and pro-IL-18 into active forms	Attenuated NLRP3 inflammasome activation and secretion of IL-1β
Higher leptin concentration in serum → growth of EAT → secretion of pro-inflammatory mediators	Reduced EAT volume, decreased leptin, PAI-1, IL-6, and TNF-α concentrations

SGLT2, sodium-glucose cotransporter-2; GLUT9, glucose transporter 9; SIRT1, sirtuin-1; XO, xanthine oxidase; FFA, free fatty acid; ROS, reactive oxygen species; β-OHB, β hydroxybutyrate; NLRP3, nod-like receptor family pyrin domain containing 3; IL, interleukin; EAT, epicardial adipose tissue; PAI-1, plasminogen activator inhibitor-1; TNF-α, tumor necrosis factor α.

**Table 3:** A summary of pathophysiological knowledge and effects of SGLT2 inhibition on endothelial function.

Pathophysiological Mechanism	Change Associated with SGLT2 Inhibition
Increased ROS levels → attenuated phosphorylation of eNOSSer1177	Increased phosphorylation of eNOSSer1177
Decreased NO bioavailability	Suppressed proliferation of vascular smooth muscle cells
Overexpression of adhesion molecules and pro-inflammatory cytokines	Decreased expression of pro-inflammatory biomarkers—Iba1, IL-6, TNF-α, IL-1β, hs-CRP, and MCP-1
Increased ROS levels → activation of transcription factors such as NF-κB	Decreased production of ROS

SGLT2, sodium-glucose cotransporter-2; ROS, reactive oxygen species; eNOS, endothelial nitric oxide synthase; NO, nitric oxide; Iba1, ionized calcium-binding adaptor molecule 1; IL, interleukin; TNF-α, tumor necrosis factor α; hs-CRP, high-sensitivity C-reactive protein; MCP-1, monocyte chemoattractant protein-1; NF-κB, nuclear factor kappa B.

## Data Availability

No new data were created or analyzed in this study. Data sharing is not applicable to this article.
